# Plasma Aβ42/GFAP Predicts Cognitive Health in Younger Adults from Diverse Latin American Countries

**DOI:** 10.1093/geroni/igaf122.4269

**Published:** 2025-12-31

**Authors:** Erica Diminich

**Affiliations:** California State University, Dominguez Hills, Dominguez Hills, California, United States

## Abstract

Latino adults face nearly twice the risk of Alzheimer’s disease (AD) compared to non-Latino Whites, yet remain underrepresented in plasma biomarker research. This study addresses a critical gap by examining blood-based biomarkers of AD-related neuropathology and brain health with cognitive performance in younger adults using community placed research to advance health equity in AD research. Objectives were to determine associations between plasma AD biomarkers with cardiometabolic factors and validate these biomarkers with NIH Toolbox cognitive assessments in community-dwelling Latino adults at early midlife. We enrolled 164 foreign-born Latino adults (mean age 48.43) in a community-placed research study. Plasma biomarkers were quantified using Simoa technology. The NIH Toolbox assessed six cognitive domains. Spearman correlations and multivariable linear regression models examined biomarker-cognition associations, controlling for demographics and cardiometabolic factors. Community engagement successfully recruited 98% monolingual Spanish speakers and 47% Central American participants—populations typically excluded from traditional studies. Blood biomarkers demonstrated significant age-related increases and cognitive associations: GFAP inversely correlated with executive function (ρ=-0.37, p < 0.001) and global cognition (ρ=-0.27, p = 0.006). Novel Aβ42/GFAP ratio positively predicted executive function (ρ = 0.33, p < 0.001), episodic memory (ρ = 0.25, p = 0.01), and processing speed (ρ = 0.22, p = 0.03). This work establishes the feasibility and rigor of community placed AD biomarker research in at-risk populations. Findings support the development of culturally responsive recruitment protocols, inform early intervention studies, and provide evidence for health equity approaches that can guide policy for inclusive clinical trial design and community placed early detection and prevention programs.

